# Topical therapeutic corneal and scleral tissue cross-linking solutions: *in vitro* formaldehyde release studies using cosmetic preservatives

**DOI:** 10.1042/BSR20182392

**Published:** 2019-05-03

**Authors:** Anna Takaoka, Kerry Cao, Eric M. Oste, Takayuki Nagasaki, David C. Paik

**Affiliations:** Department of Ophthalmology, Columbia University, Vagelos College of Physicians and Surgeons, New York, NY, U.S.A

**Keywords:** chromotropic acid, cornea, diazolidinyl urea, DMDM hydantoin, formaldehyde releasers, sclera, sodium hydroxymethylglycinate, tissue cross-linking

## Abstract

Our recent tissue cross-linking studies using formaldehyde releasers (FARs) suggest that corneal and scleral tissue strengthening may be possible without using ultraviolet irradiation or epithelial removal, two requirements for the photochemical method in widespread clinical use. Thus, the present study was carried out in order to better understand these potential therapeutic solutions by studying the effects of concentration, pH, buffer, time, and tissue reactivity on formaldehyde release of these FARs. Three FARs, sodium hydroxymethyl glycinate (SMG), DMDM, and diazolidinyl urea (DAU) were studied using a chromotropic acid colorimetric FA assay. The effects of concentration, pH, and buffer were studied as well as the addition of corneal and scleral tissues. The main determinant of release was found to be dilution factor (concentration) in which maximal release was noted at the lowest concentrations studied (submillimolar). In time dependent studies, after 60 min, FA levels decreased by 38% for SMG, 30% for DMDM, and 19% for DAU with corneal tissue added; and by 40% for SMG, 40% for DMDM, and 15% for DAU with scleral tissue added. We conclude that concentration (dilution factor) was found to be the most important parameter governing the percent of FA released.

## Introduction

Ultraviolet-A riboflavin photochemical corneal cross-linking (known as ‘CXL’) has created a new option in the field of corneal therapeutics. In the short period since its development, CXL has been proven both effective and safe in stabilizing patients with keratoconus (KC) [1–4] and post-LASIK keratectasia [5,6], and is becoming standard of care throughout the world and has recently received FDA approval for use in the United States. The cross-linking procedure effectively halts the progression of KC and can be accompanied by an improvement in both corneal curvature (i.e., flattening or ‘normalization’ of astigmatism) and/or visual acuity [5–9]. Furthermore, the indications are ever-expanding to include investigations into treating bullous keratopathy, corneal infections and melts, pellucid marginal degeneration, and in combination with refractive surgery (either pre- or postprocedure) [10–13]. As good as it is, CXL has limitations and thus developing a topically delivered adjunct (or alternative) method of inducing corneal tissue cross-linking could improve patient care. Potential benefits include the following: (1) eliminate the need for ultraviolet irradiation, (2) self-administration, (3) more homogeneous cross-linking throughout the extent of the corneal stroma, and (4) the possibility of modulation of degree of cross-linking effect.

Ever since their discovery, cross-linking agents have sparked much interest in their applications, resulting in their use for a variety of purposes in industry and the biomedical sciences. We recently reported on the *in vitro* testing of a group of potential cross-linking compounds in widespread use as chemical preservatives in cosmetics and other personal care products (PCPs) known as formaldehyde releasing agents (or FARs) [14]. FARs are a versatile class of compounds that could potentially act as an adjunct or even an alternative to CXL treatment. These compounds have received much attention and are found in a wide variety of man-made products; mostly those from the textile industry as well as personal care products, such as toilet deodorizers, shampoos, conditioners, and plasticizers [15]. They also serve as popular and attractive starting compounds or chemical intermediates in the synthetic route of organic compounds used in industry [16]. This includes polymer chemistry, where they act as formaldehyde donors and are used in the production of resins, plastics, polyesters, and polyurethane products to cross-link compounds such as urea, melamine, phenols, and resorcinol [17]. Such widespread use of FARs (which include nitroalcohols) has naturally led to extensive study into the acute toxicity, teratogenicity, and mutagenicity/carcinogenicity profiles of these compounds, which are all favorable [15].

One of the most common uses for FARs is in cosmetics as preservatives. In the United States and some countries in Europe, DAU, and DMDM are two FARs present in the highest frequencies followed by SMG [18,19]. In addition to use as a cosmetic preservative, DMDM is known to possess antimicrobial properties against fungi, yeast, and Gram-positive and Gram-negative bacteria [20]. SMG has additional application in cleaning/washing agents, rinsing agents, and as a neutralizing agent for acids/acrylics polymers [21]. Although there are many compounds that are suitable for industrial and commercial cross-linking purposes, there are only a limited number of compounds that can be considered for use *in vivo*. This is due to additional concerns that would normally be irrelevant in the context of *in vitro* studies and include efficacy under physiologic pH and temperature, permeability, coloration, and effects on light transmission, and cell toxicity.

Using FARs commonly found in personal care products and the textile industry as cross-linking agents is justified by several reasons. First, their applications in commonly used products suggest that these compounds can be used for therapies. Second, many FARs possess antimicrobial activity, which would be useful when considering their application in live subjects, such as patients with keratoconus and post-LASIK keratectasia. Third, these compounds are commercially available and can be readily obtained. Although FARs have been studied in the past for their preservative capabilities (i.e., shelf-life) and industrial cross-linking capabilities, the specific cross-linking capability of these FARs with the intended use for *in vivo* tissue cross-linking, has not been explored previously.

The current study is an extension of our previous studies [14,22,23] in which we now study the factors governing the release of formaldehyde (FA), the key compound responsible for generating cross-links between collagen molecules. Using a simple, traditional colorimetric assay adapted to a 96-well plate format, we compared three commercially available FARs for their respective FA-releasing ability and reactivity with corneal tissue substrate, including sodium hydroxymethylglycinate (SMG), DMDM, and diazolidinyl urea (DAU), three potentially clinically useful compounds for *in vivo* use. The data presented were able to show that the release of FA is primarily dependent upon concentration and not pH dependent.

## Materials and methods

### Chemicals

Formaldehyde solution (36.8–38%), Diazolidinyl urea (N-hydroxymethyl-N-(1,3-di(hydroxymethyl)-2,5-dioxoimidazolidin-4-yl)-N’-hydroxy-methylurea [DAU]), chromotropic acid disodium salt dehydrate (technical grade), sulfuric acid (99.99%), 1N hydrochloric acid, and sodium bicarbonate were purchased from Sigma–Aldrich Corp. (St. Louis, MO, U.S.A.). SMG was obtained from Tyger Scientific Inc. (Ewing, NJ, U.S.A.). DMDM hydantoin (DMDM) was obtained from Oakwood Products Inc. (West Columbia, SC, U.S.A.). All chemical solutions and buffers were prepared fresh using Millipore water (double distilled, deionized water, ρ = 18.2 MΩcm at 25°C) on the day of the experiment.

### pH of FAR solutions

SMG, DMDM hydantoin (DMDM), and DAU solutions were dissolved using Millipore water, 0.1N HCl or 0.1M sodium bicarbonate solution to appropriate concentrations at each day of the experiment. pH of solutions was measured using a pH meter (Orion Star™ A221, Thermo Fisher Scientific Inc. Waltham, MA, U.S.A.).

### Formaldehyde release assay

The standard formaldehyde solutions and the aqueous chromotropic acid solutions were freshly prepared on each day that calibration lines were determined. Formaldehyde solution (36.8–38%) was diluted to make a stock formaldehyde solution of 10 µg/ml. Stock solution was then further diluted to prepare 5, 2.5, 1.25, and 0.625 µg/ml standard formaldehyde solutions. For the solvent comparison experiment, standard solution was prepared using Millipore water, 0.1N HCl or 0.1M sodium bicarbonate solution. The basic National Institute for Occupational Safety and Health (NIOSH) procedure was followed for the determination of calibration lines for standard formaldehyde solutions and the measurement of formaldehyde release in FAR solutions with slight modifications [24]. Briefly, 200 µl of each standard formaldehyde solution and FAR solutions at appropriate concentration was aliquoted into clean Eppendorf tubes. To each of these solutions, 30 µl of 5% chromotropic acid solution was added and thoroughly mixed by vortexing. Then 300 µl of concentrated sulfuric acid (99.99%) was added. The mixtures were thoroughly vortexed. Tubes were then placed in a water bath (80–90°C) for 1 h. The tubes were cooled down to room temperature and vortexed before spectrophotometric determinations. Absorbance was measured at 570 nm by using a spectrophotometer (ELx800; Bio-Tek Instruments, Inc., Winooski, VT, U.S.A.).

### Time course experiments using corneal and scleral tissue added to the FAR solution

At least 4 ml of standard formaldehyde solutions (10, 5, 2.5, 1.25, and 0.625 µg/ml), and stock FAR solutions were prepared freshly at each day of the experiment. Total 80–100 mg of porcine corneal or sclera tissue (previously frozen, purchased from Clements Food Group, Hatfield, PA, U.S.A.) was incubated with 2 ml FAR in water. At every time point (0, 20, 40, and 60 min) after tissue was in contact with the solution, 2 × 200 µl aliquots were taken and subjected to chromotropic formaldehyde release assay (refer to the previous formaldehyde release assay section). The reactions were carried out in sealed tubes and the opening of tubes was minimized in order to limit any potential FA release to the air.

## Results

**Scheme 1:**
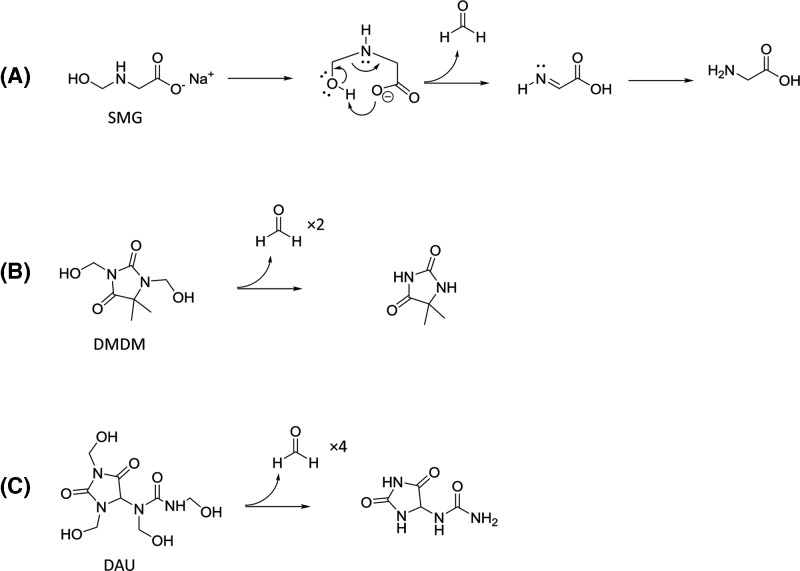
Formaldehyde release/decomposition of FAR compounds Known/proposed chemistry of (**A**) SMG decomposition into formaldehyde and glycine, (**B**) DMDM into formaldehyde and dimethylhydantoin, and (**C**) DAU into formaldehyde and allantoin.

Due to their widespread use as cosmetic preservatives, their safety with regard to mutagenicity/carcinogenicity has been well studied. That being said, the safety of FAR intended for the corneal and scleral cross-linking has been limited to our initial and ongoing live rabbit studies [23,25]. Table 1 and Scheme 1 summarize the characteristics of FARs used in the present study, including the theoretical FA release, partition coefficient, max allowed concentration in cosmetics, mutagenicity, and LD50 toxicity. Initial *ex vivo* rabbit cross-linking studies suggested that SMG, DMDM, and DAU were the FARs which demonstrated the highest cross-linking efficacy and also thought to be the most clinically useful based on size and lower toxicity profiles (data not shown) [14]. Therefore, formaldehyde release experiments in the present study, evaluating pH, buffer dependency, and dilution factor, were limited to these three compounds.

**Table 1:** Formaldehyde releasers evaluated in the present study and their known characteristics

Name	MW	Structure	Mole of FA released per 1 mole of FAR	Predicted octanol/water partition coefficient, LogP	Max allowed concentration (mM)	Mutagenicity	Toxicity
SMG [CAS # 70161-44-3]	127.07	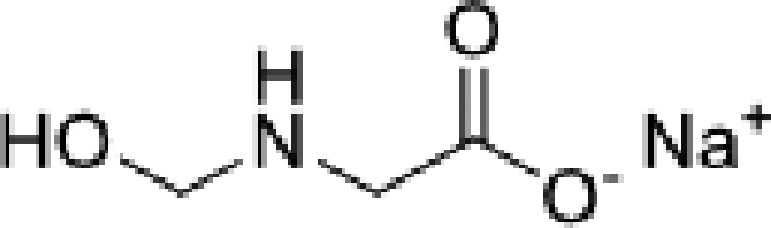	1	−1.197 [41]	39.06 [42]	N [43]	LD50 oral, rat, 2100 mg/kg; LD50 dermal, rabbit, >2000 mg/kg [43]
DMDM hydantoin (DMDM) [CAS # 6440-58-0]	188.18	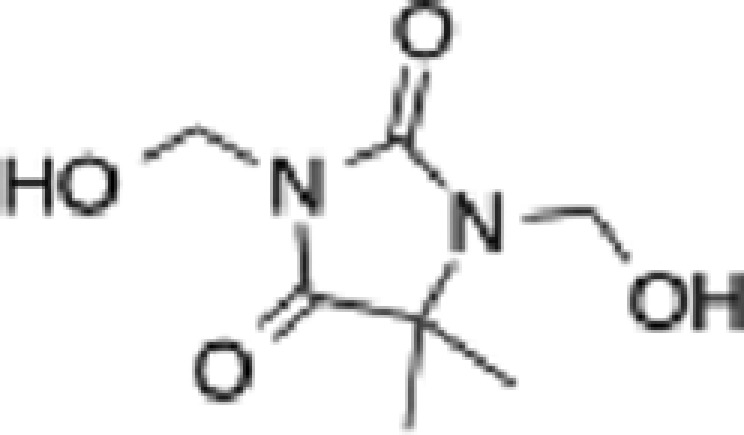	2	−2.3 [44]	31.88 [42]	N [45]	LD50 oral, rat, 3720 mg/kg; LD59 oral, rat, >2000 mg/kg [45]
DAU [CAS # 78491-02-8]	278.22	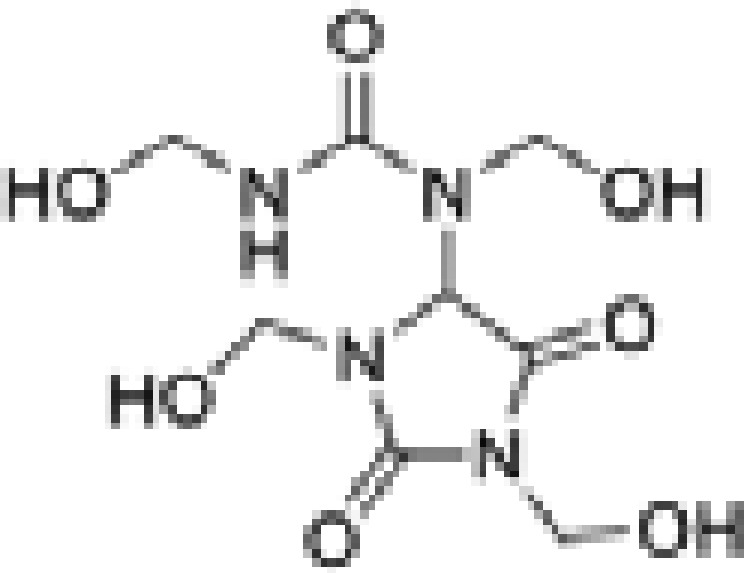	4	−5.398 ± 0.866 [46]	17.97 [42]	N [47]	LD50 oral, rat, 2600 mg/kg; LD50 dermal, rabbit, >2000 mg/kg [48]

### Determination of FAR pH in various solutions

**Figure 1:**
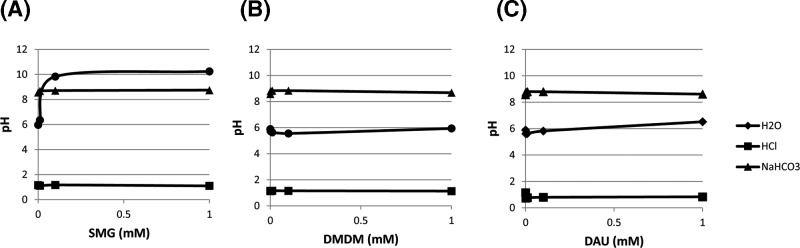
pH of FAR (A: SMG, B: DMDM, C: DAU) solutions measured in various solvents (unbuffered water, 0.1N HCl, and 0.1M NaHCO_3_) FAR solution was made by dissolving appropriate amount of solid in unbuffered water, 0.1N HCl, and 0.1M NaHCO_3_. The pH was determined with 3 ml aliquot of each solution in a falcon tube.

Figure 1A–C show the effect of active FAR compounds on pH in three different solutions, namely unbuffered water, 0.1N HCl and 0.1M NaHCO_3_. All except for SMG in unbuffered water did not have impact on solution pH, at any concentration. SMG in unbuffered water, however, seem to have caused pH to fluctuate, especially at the lower concentration. At high concentration of SMG, pH of unbuffered water reaches as high as pH of 10. DMDM and DAU were able to keep the unbuffered water at around a constant pH range of 6. Unlike the other FARs, SMG is not a cyclic compound and it is this structural difference, namely the greater difficulty of a linear molecule to stabilize the negative charge (concentrated on the nitrogen atom after the liberation of FA) that may contribute to its relatively high basicity (Scheme 1).

### Formaldehyde release assay

The amount of FA released in each solution was determined by the chromotropic acid method. Chromotropic acid is one of the reagents which reacts with FA with high selectivity [24,26]. Though, the chemistry of the reaction of chromotropic acid with FA with sulfuric acid has not been completely solved, it is believed that the reaction is carried out in two steps. First, 2 moles of chromotropic acid reacts with 1 mole of FA to form a series of diazotized derivatives of aromatic compounds, in which para, para-quinoidal adduct was the most often quoted structure. Then the sulfuric acid oxidizes p-quinoidal adduct to mono-cationic dibenzoxanthylium, which absorbs light in the range of 570–580 nm [26].

### Formaldehyde release by SMG in various solutions (Figure 2)

SMG is an amine based formaldehyde releaser, and 1 mol of SMG releases 1 mol of FA and results in decomposition into glycine. At a concentration lower than 0.3 mM, SMG decomposes into FA and glycine at any pH.

**Figure 2:**
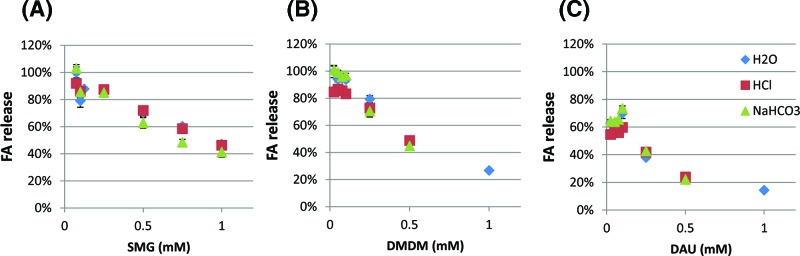
Effect of concentration (dilution factor) and pH on FA release for three FAR compounds (**A**) SMG, (**B**) DMDM, and (**C**) DAU. A colorimetric FA assay, chromotropic acid assay was used for the determination of free formaldehyde in FAR solutions. For SMG, concentrations ranging from 0.075 to 1 mM were assayed in three different solutions, a very acidic 0.1N HCl (pH = 2), a 100 mM NaHCO_3_ solution maintaining a pH close to 8.5, and an unbuffered solution made up in H_2_O. For DAU and DMDM, concentrations ranging from 0.025 to 1 mM were assayed in the three different solutions. The amount of formaldehyde released into solution at time = 10 min for each compound was expressed as a percentage of that predicted, based on the theoretical complete release of formaldehyde. (**A**) SMG releases 1 mole/mole, (**B**) DMDM releases 2 moles/mole and (**C**) DAU releases 4 moles/mole.

Unlike the study by Emeis et al., our test was done at a lower concentration and we did not observe complete dissociation at 1 mM, the highest concentration we tested. At 1 mM, the equilibrium was reached and only 50% of FA was released. Buffer types/pH did not affect the release percentage. This could be clinically relevant as SMG has known storage and shelf life properties based on monitoring of a very concentrated solution.

### FA release by DMDM and DAU in various solutions (Figure 2)

DMDM and DAU are also known to reliably decompose and we have found DMDM and DAU to release FA in water without any addition of bicarbonate buffer. According to the structures of DMDM and DAU, DMDM can release 2 moles of FA and DAU can release 4 moles of FA. Our FA assay showed that DMDM at 0.5 mM concentration released 48% of its releasable FA in water, 49% in HCl, and 45% in NaHCO_3_. When DMDM was further diluted to 0.1 mM or below, DMDM seemed to reach complete dissociation. When DAU was dissolved to 1 mM concentration in water, it only released less than 20% of releasable FA and reached equilibrium. At 0.25 mM, DAU released 38% (in water), 42% (in HCl) and 43% (in NaHCO_3_) of releasable FA. The highest % of free FA (ca. 60%) was detected when DAU was diluted to 0.1 mM or below. There was no significant difference in the release % in different solutions.

### Time course experiments using corneal and scleral tissue added to the FAR solution (Figures 3 and 4)

**Figure 3:**
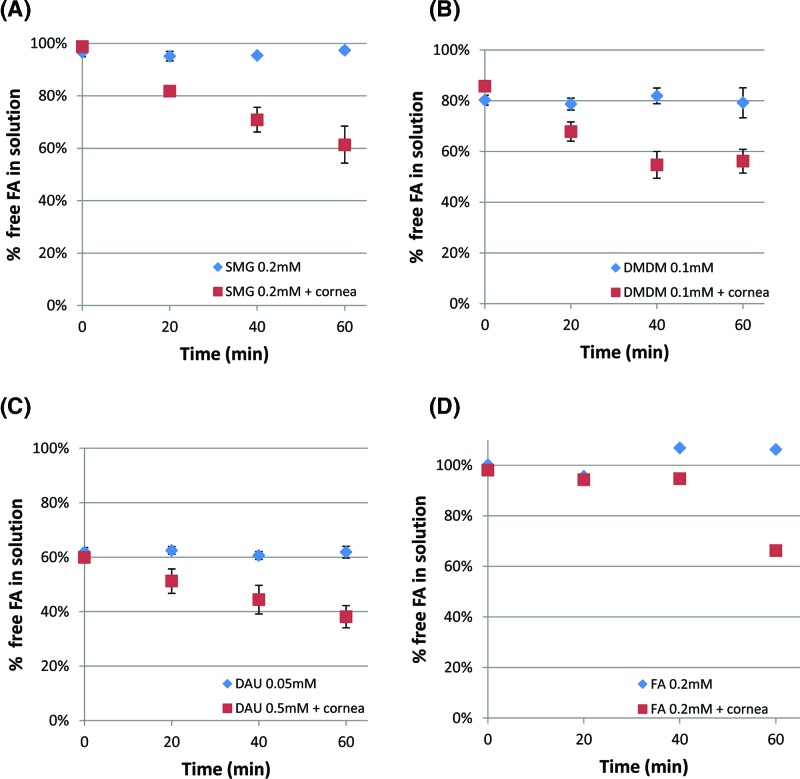
Change in FA level was measured over 0–60 min at various FAR concentrations incubated with porcine corneal tissue (40–50 mg/ml) FAR concentrations used were as follows: (**A**) SMG at 0.2 mM, (**B**) DMDM at 0.1 mM, (**C**) DAU at 0.05 mM, and (**D**) FA at 0.2 mM. All solutions were made in unbuffered water. At t = 0, 20, 40, 60 min, an aliquot was taken from the reaction mixture, and free FA in the solution was measured using the chromotropic acid assay. FA levels decreased in a time dependent manner. The levels at 60 min decreased by (**A**) 38% for SMG, (**B**) 30% for DMDM, (**C**) 19% for DAU, and (**D**) 32% for FA. The difference in levels between tissue containing solutions and control at 60 min were all statistically significant at *P*<0.05.

**Figure 4:**
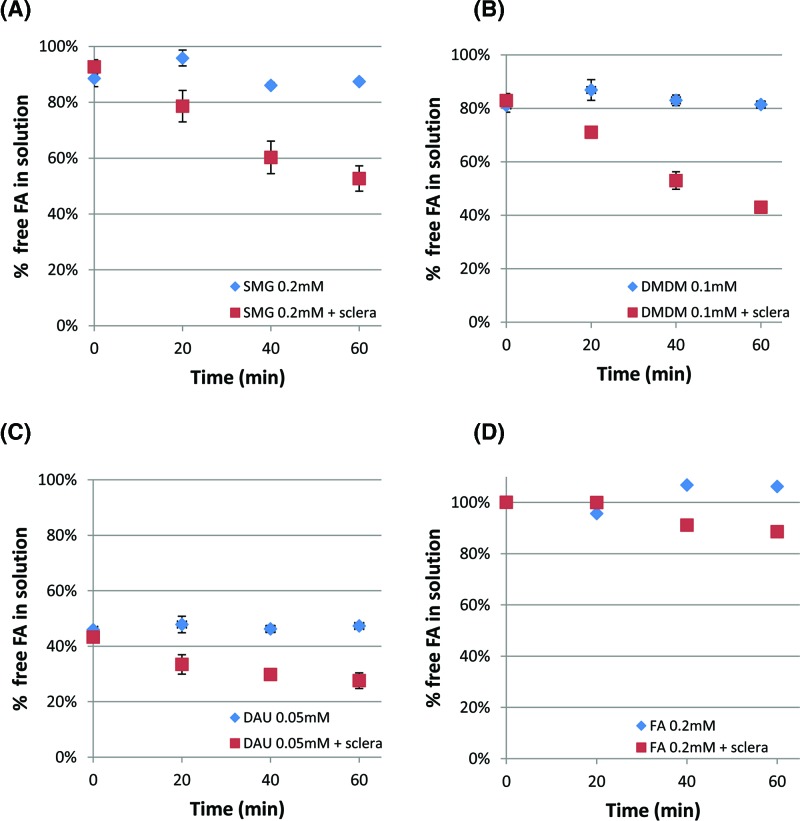
Change in FA level was measured over 0–60 min at various FAR concentrations incubated with porcine scleral tissue (40–50 mg/ml) FAR concentrations used were as follows: (**A**) SMG at 0.2 mM, (**B**) DMDM at 0.1 mM, (**C**) DAU at 0.05 mM, and (**D**) FA at 0.2 mM. All solutions were made in unbuffered water. At t = 0, 5, 20, 40, 60 min, an aliquot was taken from the reaction mixture, and free FA in the solution was measured using the chromotropic acid assay. FA levels decreased in a time dependent manner. The levels at 60 min decreased by (**A**) 40% for SMG, (**B**) 40% for DMDM, (**C**) 15% for DAU, and (**D**) 11% for FA. The difference in levels between tissue containing solutions and control at 60 min were all statistically significant at *P*<0.05.

In the final set of experiments, we evaluated changes in FA levels when corneal tissue substrate is introduced into the mixture, similar to that which would occur *in vivo*. Porcine corneal and scleral tissues (previously frozen, cut pieces) were utilized as substrates and incubated with FAR solution up to 60 min. At several time points, aliquots were taken from the reaction mixture and subjected to the formaldehyde release assay. The results are shown in Figures 3 and 4. The level of FA at 0 min represents the level of FA in the solution before the substrate was added. At 20 min of SMG 0.2mM incubated with tissue substrates, the level of free FA in the solution was reduce by approximately 20% compared with control (SMG solution without tissue substrate), and the level of free FA in the solution continued to decrease for the time period studied. Similar trends to SMG 0.2 mM were obtained using DMDM at 0.1 mM, which theoretically can release 0.2 mM of FA. When incubated with corneal substrate, the amount of free FA released from DMDM decreased from 86 to 56% within an hour, while the free FA of the control solution without tissue substrate did not change. When incubated with scleral substrate, free FA released from DMDM decreased from 83 to 43%. When 0.05 mM DAU was incubated with tissue substrates, free FA level decreased from 61 to 41% in a sample incubated with corneal tissue and from 43 to 28% with scleral tissue. Slight differences between corneal versus scleral tissue experiments could be attributed to the differences in reactivity between the tissue samples. It should be remembered that because DMDM (1:2) and DAU (1:4) produce greater amounts of FA per mole than SMG (1:1), the lower percentage differences noted for DMDM and DAU as compared with SMG (Figures 3 and 4), do not necessarily reflect a lower amount of FA consumed by the tissue substrate. In other words, for example, a 40% decrease in SMG (1:1) would be equivalent to 20% decrease in DMDM (1:2), when considering absolute amounts of FA. Finally, FA levels of FAR solutions without tissue substrate added did not change over the course of 60 min. This suggests that the tissue substrate added provided reactive sites for FA. Such FA protein reactions are well known to occur and are discussed in greater detail below.

## Discussion

Our previously reported screening for more effective, yet safe, chemical agents that can induce tissue cross-linking has yielded several potential candidates, including SMG, DMDM, and DAU [14]. Thus, due to their demonstrated efficacy in inducing tissue cross-linking in the cornea and sclera in that previous study, we chose to focus our analysis of FA release on these same compounds, utilizing a simple colorimetric assay for quantitation. There are other, more sophisticated methods for measuring formaldehyde and following the decomposition of FARs such as ^1^H and ^13^C NMR. However, specialized analytical instrumentation is necessary and may not be available to many labs, particularly in institutions with more limited resources. Also, when the dilution is the crucial factor for the FA release and when it is required to work with such diluted solutions, NMR measurement is not feasible. Thus, we believe that this is a good starting point for trying to understand how the FARs may function in *in vivo* systems. Consistent with prior literature, concentration (or dilution) was determined to be the primary factor governing formaldehyde release [27].

The equilibrium of FA for these three compounds has been studied and reported previously using ^13^C NMR by Emeis et al (2007). In that study, although the concentration ranges tested were significantly higher than those reported in our study, the authors found that concentration was a major factor which drives the release of FA. The reason for studying this concentration range in our study (Figure 2), which is much lower than those reported by Emeis et al., was in order to evaluate FA release at concentrations that would be expected intrastromally to result from topical application of a (‘max allowed’) concentration. In other words, since it is known that approximately 1% or less of a topically applied eye drop will reach the stroma [28,29], this is the concentration range that we will be producing intrastromally. For example, for SMG, a 40 mM ( = 0.5% max allowed) eye drop will allow for approximately 0.4 mM intrastromal concentrations. For DMDM, the levels would be approximately 32 mM ( =0.6% max allowed) for a 0.32 mM intrastromal concentration and for DAU, the level would be approximately 18 mM ( =0.5% max allowed) for a 0.18 mM instrastromal concentration.

DAU is an allantoin derivative, where allantoin reacts with four equivalents of formaldehyde under basic conditions to form the parent compound [19]. Dilution encourages the decomposition reaction, overcoming possible steric interference, and facilitating the separation of the formaldehyde moiety from the mother compound. Lehmann et al. demonstrated that DAU exists as a mixture of isomers, with ‘compound BHU’ (1-(3,4-bis-hydroxymethyl-2,5-dioxo-imidazolidin-4-yl)-1,3-bis-hydroxymethyl-urea) as the dominant form (30–40%) [30]. It is hypothesized that the remainder consists of many polymers of allantoin-formaldehyde condensation products. It is conceivable that the impurity of the DAU sample could result in inconsistent reaction thermodynamics and kinetics.

Regarding FA release, DAU was reported for two concentrations by Emeis et al., approximately 18 mM and approximately 7 mM. Higher levels of free FA occurred at the highest pH 9 (compared with pH 6 and pH 4). As well, higher FA was detected at the lower concentration (7 mM), once again consistent with a concentration dependent release effect. Of note, the maximum amount of free FA detected was approximately 2× of the starting material which is significantly less than the expected amount based on DAU’s chemical structure, which suggest that 4 moles of FA can be theoretically released from each mole of DAU [31]. This lower than expected release was also noted in our study (Figure 2C). One explanation for the low amount of free FA release from DAU is that the experimental environment in the first two sets of experiments was in a ‘closed system’ in that there is no substrate available for FA modification. Thus, the reaction would be approaching equilibrium during which time it is possible that the liberated FA can react with other reaction products of the FAR decomposition. This includes the reverse reaction to regenerate starting material, resulting in a lower quantity of free formaldehyde. This fact notwithstanding, DAU consistently released the most formaldehyde out of the selected FARs of the present study, since it possesses a much higher molar formaldehyde releasing capacity than the other FARs, DMDM, and SMG.

DMDM is a hydantoin with a theoretical yield of two FA moieties. DMDM has one of the higher maximum allowed concentrations of the FARs used in the present study (0.6%). Regarding FA release, DMDM is a smaller size molecule and has a theoretical yield of two moles of FA per mole of DMDM. For DMDM at concentrations from approximately 3 mM to approximately 1.3 M a more alkaline pH (8.5–9 as compared with pH 6–6.5 and pH 4–4.5) and a lower concentration favored higher levels of free FA, consistent with the release characteristics of DAU, which is also increased at lower concentrations [31]. Once FA is liberated from either of the nitrogen atoms of the five-membered ring, the resulting negative charge on the nitrogen atom is delocalized into the π-system provided by both of the adjacent carbonyl moieties. The formation of intramolecular hydrogen bonds between local DMDM molecules stabilizes any additional negative charge.

Another low molecular weight FAR, SMG, demonstrates similar behavior to DMDM with regards to near total theoretical formaldehyde release. SMG has a theoretical yield of one mole of FA per mole of SMG. In spite of this lower ratio when compared with the other FARs of the present study, SMG appears to release FA more readily than DAU and behaved differently from the other FARs in certain regards. Solutions of SMG in water tend to be highly alkaline (Figure 1) but can be modulated downward with the addition of bicarbonate buffer (approximately pH 9.2) which buffers maximally in the pH 9.2–10.8 range. The solution pH can be lowered significantly further into the acidic range using 0.1N HCl without having an effect on the formaldehyde yield (Figure 2) which is in some contrast with the findings from Emeis et al. that indicate SMG releases more formaldehyde under acidic conditions. The degradation of SMG seems to be favored compared with other compounds that reaches equilibrium. Further studies are required to elucidate the details of this trend and can include ^1^H NMR, ^13^C NMR, and HPLC applications.

The reactions of free FA and related preparations (i.e., paraformaldehyde) have been studied in depth owing to their importance as tissue cross-linking agents for histologic purposes. Known reactivity of formaldehyde with tissue for histology are primarily with proteins and avoid carbohydrates, lipids, and nucleic acids, which are trapped in cross-linked matrix and are not chemically altered unless fixation is prolonged [32]. Reaction with DNA nucleotide bases such as adenine and cytosine have been reported to occur. However, the reactions require high concentration of free FA and relatively long incubation times, making them less likely to be relevant in our types of reactions [33].

Several reactions have been shown to occur between formaldehyde and free amino acids, and include reaction with lysine, asparagine, threonine, cysteine, histidine, and tryptophan [34]. In addition, stable intermolecular cross-links can form between the sulfydryl group of cysteines and side chains of lysine, arginine, asparagine, and glutamine. In reactions with proteins, formaldehyde reactions with several different proteins have been studied and include bovine serum albumin (BSA) [35], insulin [36], and cottonseed protein [37], for example. Of these, BSA has been studied the most extensively. Both acid-labile and acid-stable products can form and include hydroxymethyl adducts and methylene bridges between combinations of lysine, arginine, asparagine, glutamine, and tyrosine. Reactions with insulin have been studied by Metz et al. (2006) and indicated reactivity with lysine, arginine, and tyrosine. Similarly, reactions with cottonseed protein involve primarily lysine and tyrosine [37]. The reactions with collagen and formaldehyde have been reported to take place at ε-amino groups of lysine residues [38], and further form cross-links with the nitrogen atom of a peptide linkage [34,39,40]. These studies, while helpful in understanding the chemical moieties that may be involved in the FAR TXL reactions, do not necessarily predict the types of cross-links and protein modification that may actually be induced by FARs since the formaldehyde is likely to be released locally and at significantly lower concentration than using preparations of free formaldehyde and its polymers.

Although no attempt was made in the present study to identify specific reaction sites or products of the reactions (either cross-link or non-cross-link adducts), lysines and hydroxylysines, are likely sites since these residues are widely involved in collagen cross-linking and non-cross-linking reactions, both enzymatic (lysyl oxidase mediated) and non-enzymatic (i.e., non-enzymatic glycation, non-enzymatic nitration, and oxidations).

## Abbreviations

BSA: bovine serum albumin
CXL: corneal cross-linking
DAU: diazolidinyl urea
DMDM: DMDM hydantoin
FA: formaldehyde
FAR: FA releasing agent
KC: keratoconus
PCP: personal care product
SMG: sodium hydroxymethylglycinate
TXL: tissue cross-linking

## References

[1] Wollensak G., Spoerl E. and Seiler T. (2003) Riboflavin/ultraviolet-a-induced collagen crosslinking for the treatment of keratoconus. Am. J. Ophthalmol. 135, 620–627 10.1016/S0002-9394(02)02220-1 12719068

[2] Phillips J.R., Khalaj M. and McBrien N.A. (2000) Induced myopia associated with increased scleral creep in chick and tree shrew eyes. Invest. Ophthalmol. Vis. Sci. 41, 2028–2034 10892839

[3] Caporossi A., Baiocchi S., Mazzotta C., Traversi C. and Caporossi T. (2006) Parasurgical therapy for keratoconus by riboflavin-ultraviolet type A rays induced cross-linking of corneal collagen: preliminary refractive results in an Italian study. J. Cataract Refract. Surg. 32, 837–845 10.1016/j.jcrs.2006.01.091 16765803

[4] Wollensak G. (2006) Crosslinking treatment of progressive keratoconus: new hope. Curr. Opin. Ophthalmol. 17, 356–360 10.1097/01.icu.0000233954.86723.25 16900027

[5] Hersh P.S., Greenstein S.A. and Fry K.L. (2011) Corneal collagen crosslinking for keratoconus and corneal ectasia: One-year results. J. Cataract Refract. Surg. 37, 149–160 10.1016/j.jcrs.2010.07.030 21183110

[6] Hafezi F., Kanellopoulos J., Wiltfang R. and Seiler T. (2007) Corneal collagen crosslinking with riboflavin and ultraviolet A to treat induced keratectasia after laser in situ keratomileusis. J. Cataract Refract. Surg. 33, 2035–2040 10.1016/j.jcrs.2007.07.028 18053900

[7] Raiskup-Wolf F., Hoyer A., Spoerl E. and Pillunat L.E. (2008) Collagen crosslinking with riboflavin and ultraviolet-A light in keratoconus: long-term results. J. Cataract Refract. Surg. 34, 796–801 10.1016/j.jcrs.2007.12.039 18471635

[8] Wittig-Silva C., Whiting M., Lamoureux E., Lindsay R.G., Sullivan L.J. and Snibson G.R. (2008) A randomized controlled trial of corneal collagen cross-linking in progressive keratoconus: preliminary results. J. Refract. Surg. 24, S720–725 10.3928/1081597X-20080901-15 18811118

[9] Vinciguerra P., Albe E., Trazza S. (2009) Refractive, topographic, tomographic, and aberrometric analysis of keratoconic eyes undergoing corneal cross-linking. Ophthalmology 116, 369–378 10.1016/j.ophtha.2008.09.048 19167087

[10] Meek K.M. and Hayes S. (2013) Corneal cross-linking - a review. Ophthalmic Physiol. Opt. 33, 78–93 10.1111/opo.12032 23406488

[11] Wollensak G., Aurich H., Wirbelauer C. and Pham D.T. (2009) Potential use of riboflavin/UVA cross-linking in bullous keratopathy. Ophthalmic Res. 41, 114–117 10.1159/000187630 19122475

[12] Al-Sabai N., Koppen C. and Tassignon M.J. (2010) UVA/riboflavin crosslinking as treatment for corneal melting. Bull. Soc. Belge Ophtalmol. 13–17 21110504

[13] Makdoumi K., Mortensen J. and Crafoord S. (2010) Infectious keratitis treated with corneal crosslinking. Cornea 29, 1353–1358 10.1097/ICO.0b013e3181d2de91 21102196

[14] Babar N., Kim M., Cao K. (2015) Cosmetic preservatives as therapeutic corneal and scleral tissue cross-linking agents. Invest. Ophthalmol. Vis. Sci. 56, 1274–1282 10.1167/iovs.14-16035 25634979 PMC4338628

[15] Bollmeier A. (1996) Nitroalcohols. Kirk-Othmer Encyclopedia of Chemical Technology, John Wiley & Sons, Inc., New York

[16] Shvekhgeimer M-GA. (1998) Aliphatic nitro alcohols: synthesis, chemical transformations and applications. Russ. Chem. Rev. 67, 35–68 10.1070/RC1998v067n01ABEH000285

[17] Swedo R. (2003) Phenolic resin systems for fiber reinforced composite manufacture. Dow. Chem., assignee U.S. Patent 10,383,272, March 7, 2003

[18] de Groot A.C., Flyvholm M.A., Lensen G., Menne T. and Coenraads P.J. (2009) Formaldehyde-releasers: relationship to formaldehyde contact allergy. Contact allergy to formaldehyde and inventory of formaldehyde-releasers. Contact Dermatitis 61, 63–85 10.1111/j.1600-0536.2009.01582.x 19706047

[19] Flyvholm M.A. (2005) Preservatives in registered chemical products. Contact Dermatitis 53, 27–32 10.1111/j.0105-1873.2005.00629.x 15982228

[20] de Groot A.C., van Joost T., Bos J.D., van der Meeren H.L. and Weyland J.W. (1988) Patch test reactivity to DMDM hydantoin. Relationship to formaldehyde allergy. Contact Dermatitis 18, 197–201 10.1111/j.1600-0536.1988.tb02802.x 3378426

[21] Russell K. and Jacob S.E. (2010) Sodium hydroxymethylglycinate. Dermatitis 21, 109–110 20233550

[22] Kim M., Takaoka A., Hoang Q.V., Trokel S.L. and Paik D.C. (2014) Pharmacologic alternatives to riboflavin photochemical corneal cross-linking: a comparison study of cell toxicity thresholds. Invest. Ophthalmol. Vis. Sci. 55, 3247–3257 10.1167/iovs.13-13703 24722697 PMC4037937

[23] Kim S.Y., Babar N., Munteanu E.L. (2016) Evaluating the toxicity/fixation balance for corneal cross-linking with sodium hydroxymethylglycinate (SMG) and riboflavin-UVA (CXL) in an *ex vivo* rabbit model using confocal laser scanning fluorescence microscopy. Cornea 35, 550–556 10.1097/ICO.0000000000000743 26807905 PMC4779747

[24] Katz M., (1977) Methods of Air Sampling and Analysis, 300–307, American Public Health Association, Washington DC

[25] Zyablitskaya M., Amponin D., Takaoka A. (2018) Sodium Hydroxymethylglycinate via Eyedrop for Corneal Cross-linking in Dutchbelted Rabbits: A Comparison of 40 mM (0.5%) vs 80 mM (1%) Concentrations, The Association for Research in Vision and Ophthalmology, Honolulu, Hawaii

[26] Georghiou P.E. and Ho C.K.J. (1989) The chemistry of the chromotropic-acid method for the analysis of formaldehyde. Canadian J. Chemistry-Revue Canadienne De Chimie 67, 871–876 10.1139/v89-135

[27] Huszal S. and Chrobak R. (2010) Formaldehyde releasers as an example of insitu generation of active substances. Biocides in Synthetic Materials, Smithers Rapra Technology Ltd, Berlin, Germany

[28] Behl G., Iqbal J., O’Reilly N.J., McLoughlin P. and Fitzhenry L. (2016) Synthesis and characterization of Poly(2-hydroxyethylmethacrylate) contact lenses containing chitosan nanoparticles as an ocular delivery system for dexamethasone sodium phosphate. Pharm. Res. 33, 1638–1648 10.1007/s11095-016-1903-7 26964548

[29] Lang J.C. (1995) Ocular drug-delivery conventional ocular formulations. Adv. Drug Del. Rev. 16, 39–43 10.1016/0169-409X(95)00012-V

[30] Lehmann S.V., Hoeck U., Breinholdt J., Olsen C.E. and Kreilgaard B. (2006) Characterization and chemistry of imidazolidinyl urea and diazolidinyl urea. Contact Dermatitis 54, 50–58 10.1111/j.0105-1873.2006.00735.x 16426294

[31] Emeis D., Anker W. and Wittern K.P. (2007) Quantitative 13C NMR spectroscopic studies on the equilibrium of formaldehyde with its releasing cosmetic preservatives. Anal. Chem. 79, 2096–2100 10.1021/ac0619985 17249689

[32] Kiernan J. (2000) Formaldehyde, formalin, paraformaldehyde and glutaraldehyde: what they are and what they do. Microscopy Today 8–12

[33] Feldman M.Y. (1967) Reaction of formaldehyde with nucleotides and ribonucleic acid. Biochim. Biophys. Acta 149, 20–34 10.1016/0005-2787(67)90687-9 5625708

[34] Thavarajah R., Mudimbaimannar V.K., Elizabeth J., Rao U.K. and Ranganathan K. (2012) Chemical and physical basics of routine formaldehyde fixation. J. Oral Maxillofac. Pathol. 16, 400–405 10.4103/0973-029X.102496 23248474 PMC3519217

[35] Tome D., Kozlowski A. and Mabon F. (1985) C-13 Nmr-study on the combination of formaldehyde with bovine serum-albumin. J. Agric. Food Chem. 33, 449–455 10.1021/jf00063a031

[36] Metz B., Kersten G.F., Baart G.J. (2006) Identification of formaldehyde-induced modifications in proteins: reactions with insulin. Bioconjug. Chem. 17, 815–822 10.1021/bc050340f 16704222

[37] Marquie C. (2001) Chemical reactions in cottonseed protein cross-linking by formaldehyde, glutaraldehyde, and glyoxal for the formation of protein films with enhanced mechanical properties. J. Agric. Food Chem. 49, 4676–4681 10.1021/jf0101152 11600006

[38] Gustavson K.H. (1947) Note on the reaction of formaldehyde with collagen. J. Biol. Chem. 169, 531–537 20259086

[39] Fox C.H., Johnson F.B., Whiting J. and Roller P.P. (1985) Formaldehyde fixation. J. Histochem. Cytochem. 33, 845–853 10.1177/33.8.3894502 3894502

[40] Mason J.T. and O’Leary T.J. (1991) Effects of formaldehyde fixation on protein secondary structure: a calorimetric and infrared spectroscopic investigation. J. Histochem. Cytochem. 39, 225–229 10.1177/39.2.1987266 1987266

[41] Sigma–Aldrich, Sodium hydroxymethylglycinate [Material Safety Data Sheet]

[42] de Groot A., White I.R., Flyvholm M.A., Lensen G. and Coenraads P.J. (2010) Formaldehyde-releasers in cosmetics: relationship to formaldehyde contact allergy. Part 2. Patch test relationship to formaldehyde contact allergy, experimental provocation tests, amount of formaldehyde released, and assessment of risk to consumers allergic to formaldehyde. Contact Dermatitis 62, 18–31 10.1111/j.1600-0536.2009.01631.x 20136876

[43] Products IS, Sodium hydroxymethylglycinate [Material Safety Data Sheet]

[44] ACME-Hardesty C, DMDM hydantoin [Material safety data sheet]

[45] (1988) 1 Final report on the safety assessment of DMDM hydantoin. J. Am. Coll Toxicol. 7, 245–277 10.3109/10915818809023133

[46] SciFinder, Chemical Abstracts Service

[47] (1990) 7 FInal report on the safety assessment of diazolidinyl urea. J. Am. Coll. Toxicol. 9, 229–245 10.3109/10915819009078735

[48] Sigma-Aldrich, Diazolidinyl urea [Material Safety Data Sheet]

